# Incidence and long‐term outcome of postradiotherapy moyamoya syndrome in pediatric patients with primary brain tumors: a single institute experience in Taiwan

**DOI:** 10.1002/cam4.785

**Published:** 2016-06-05

**Authors:** Yuan‐Hung Wu, Feng‐Chi Chang, Muh‐Lii Liang, Hsin‐Hung Chen, Tai‐Tong Wong, Sang‐Hue Yen, Yi‐Wei Chen

**Affiliations:** ^1^ Department of OncologyTaipei Veterans General HospitalTaipeiTaiwan; ^2^ Institute of Public HealthNational Yang‐Ming UniversityTaipeiTaiwan; ^3^ Department of RadiologyTaipei Veterans General HospitalTaipeiTaiwan; ^4^ School of MedicineNational Yang‐Ming UniversityTaipeiTaiwan; ^5^ Neurological InstituteTaipei Veterans General HospitalTaipeiTaiwan; ^6^ Institute of Clinical MedicineNational Yang‐Ming UniversityTaipeiTaiwan; ^7^ Department of NeurosurgeryTaipei Medical University HospitalTaipeiTaiwan; ^8^ Department of NeurosurgeryTaipei Medical UniversityTaipeiTaiwan; ^9^ Department of Biomedical Imaging and Radiological SciencesNational Yang‐Ming UniversityTaipeiTaiwan

**Keywords:** Brain irradiation, complication, radiotherapy, vasculopathy

## Abstract

We aimed to investigate the incidence and long‐term outcome of moyamoya syndrome in pediatric patients with primary brain tumors after receiving cranial radiotherapy (RT) in a single institute in Taiwan. The complete medical records, medical images, and RT notes of 391 pediatric patients with primary brain tumors treated with cranial RT between January 1975 and December 2005 in Taipei Veterans General Hospital (TVGH), Taiwan, were entered into an electronic registry and reviewed. Eight (2%) cases of post‐RT moyamoya syndrome were identified in the sample of 391 patients. The median latency was 3 years post‐RT. Among the eight patients, three had craniopharyngioma, two had optic glioma, two had medulloblastoma, and one had a suprasellar astrocytoma. The prescribed physical doses of RT were in the range of 40–54 Gy. The incidence was highest in those with optic glioma (0.039/person‐year), followed by craniopharyngioma (0.013/person‐year), astrocytoma (0.003/person‐year), and medulloblastoma (0.002/person‐year). No patients died of vasculopathy. No difference in crude incidence was found between our results and those of other series. The incidence of moyamoya syndrome was diagnosis dependent, with the highest incidence among patients with optic glioma. No regional difference in incidence was found. Long‐term, stable neurological function may be achieved following timely surgical intervention.

## Introduction

Radiotherapy (RT) improves local control and overall survival in pediatric patients with brain tumors. However, cranial irradiation also increases the risk of stroke in cancer survivors. The estimated relative risk of stroke or transient ischemic attack (TIA) in pediatric cancer survivors who have received cranial RT was 8, with an incidence of 548/100,000 person‐years 1. Besides stroke and TIA, lacunar lesions, vascular occlusive disease including moyamoya syndrome, vascular malformations including aneurysm, and hemorrhage also can be induced by radiation 2. Moyamoya vasculopathy is characterized by progressive stenosis of the intracranial internal carotid arteries and their proximal branches 3. When presenting with known associated risk factors, such as prior radiotherapy, patients with moyamoya vasculopathy are said to have moyamoya syndrome. Otherwise, they are said to have moyamoya disease. Post‐RT moyamoya syndrome, although known to be radiation related, is infrequently described in the literature. Between 1971 and 2015, there have been a total of 84 reported cases of moyamoya syndrome following cranial RT, of which 76 occurred in children 4, 5, 6, 7, 8, 9, 10, 11. The long‐term clinical outcome of these patients with post‐RT moyamoya syndrome also remains unclear. In addition, ethnic and regional differences in the incidence of moyamoya disease have been noted. In Japan, moyamoya disease is the most common pediatric cerebrovascular disease, with a prevalence of about three cases per 100,000 children 12, 13, 14. In the United States, incidence ratios are 4.6 for Asians, 2.2 for African‐Americans, and 0.5 for Hispanics, compared with Caucasians 15. Incidence per 100,000 patient‐years ranged in Japan from 0.35–0.94, 0.15 in Taiwan, in the United States from 0.05 in Iowa to 0.17 in Hawaii, and 0.41 in Nanjing, China 16, 17. It is unknown whether the incidence of RT‐related moyamoya syndrome has regional or interethnic difference. In this study, we investigated the incidence and long‐term clinical outcome of moyamoya syndrome in pediatric patients with primary brain tumors treated with cranial RT.

## Materials and Methods

Taipei Veterans General Hospital (TVGH) has been dedicated to the treatment of pediatric patients with brain tumors for more than 30 years 18. With 100–150 new cases each year in Taiwan, more than 986 cases were treated in TVGH from 1975 to May 2004 19. All patients were treated and followed by the same team, comprised of pediatric neurosurgeons, pathologists, neuroradiologists, pediatric neuro‐oncologists, and radiation oncologists. To improve the efficiency of management and use of clinical data, an electronic registry system was established in 2008. According to this registry, between January 1975 and December 2005, there were 564 pediatric patients with brain tumors who received cranial RT in TVGH. However, complete RT notes and follow‐up imaging were only available in 391 cases (69.3%). The demographic data of these patients are listed in Table 1. All patients were residents of Taiwan, and none had a first‐degree relative with moyamoya disease. The median follow‐up period was 7.3 years. Forty of the patients received repeated RT of different course. Only the physical doses of the first courses are listed in the median doses reported.

**Table 1:** Patient demographic data

Diagnosis	No. of cases	Mortality	Median age (year)	Median RT dose (cGy)
Medulloblastoma	100	54	7	5130
Atypical teratoid/rhabdoid tumors	18	12	4.3	5000
PNET^a^	6	4	5.5	4842
Optic glioma (grade 1)	6	2	5.5	5069
Pilocytic astrocytoma^b^ (grade 1)	20	5	10.5	5517.5
Astrocytoma (grade 2)	19	4	10	5508
Oligodendroglioma (grade 2)	5	2	11	5613
Ependymoma (grade 2)	8	6	5.5	4673
Anaplastic astrocytoma (grade 3)	36	24	10	6237
Anaplastic ganglioglioma (grade 3)	1	0	7	6400
Anaplastic ependymoma (grade 3)	7	2	9	5300
Glioblastoma (grade 4)	17	12	10.5	5600.5
Ependymoblastoma (grade 4)	1	0	14	5260
Astroblastoma (grade 4)	1	1	8	5573
Brain stem tumor (without pathological diagnosis)	35	23	8	6573
Germinoma	66	8	13	3800
Other germ cell tumors^c^	16	4	10	5109
Meningioma	5	3	12	5804
Craniopharyngioma	24	3	9	5400
Total	391	169		

RT, Radiotherapy

a This number includes two patients with pineoblastoma.

b Other than optic glioma.

c This category includes five patients with yolk sac tumors, three patients with mature teratomas, one patient with an immature teratoma, one patient with an unclassified germ cell tumor, and six patients with mixed germ.

A post‐RT stroke or TIA occurred in 13 of the 391 patients listed in Table 1. A neuroradiologist reviewed medical imaging of these cases to determine whether any had moyamoya vasculopathy. The incidence was calculated by number of patients with post‐RT moyamoya syndrome divided by the total follow‐up person‐years for each diagnosis of a primary brain tumor. Kaplan–Meier survival analysis was used to estimate the cumulative incidence, and the log‐rank test was used to compare the cumulative incidence between different diagnoses. To compare the crude incidence of different diagnoses from other published series, a two‐tailed Fisher's exact test was used. Statistical analyses were performed using SPSS Statistics version 20 (IBM, Armonk, NY). All *P*‐values less than 0.05 were considered significant. This study was approved by the hospital's institutional review board.

## Results

Among the 13 patients with post‐RT stroke or TIA, nine were found to have moyamoya vasculopathy. One patient, a 5‐year‐old girl with optic glioma and neurofibromatosis type‐1 (NF1) was found with moyamoya vasculopathy before radiotherapy based on a postoperative MRI and was thus excluded. The remaining eight patients had received angiography for suspected moyamoya syndrome. Their clinical characteristics are listed in Table 2. The median latency was 3 years post‐RT. Of the 391 patients included, follow‐up of more than 3 years was achieved in 235 (60.1%) of them. More than 10 years follow‐up was reached in 162 (41.4%) patients.

**Table 2:** Characteristics of pediatric patients with post‐RT moyamoya syndrome (MMS)

Diagnosis	Sex; age at RT (year)	MMS latency (year)	RT dose (cGy)/site	Symptoms	NF^a^	U/B^b^	A/P^c^	Bypass^d^	Recurrent TIA/stroke	Outcome^e^
Astrocytoma	M; 15	4	5400/suprasellar	Int. gen. weakness, drowsiness for 6 months.	–	B	A	+	–	Independent
Optic glioma	M; 6	2	4000/suprasellar	Int. gen. weakness, conscious change for 1 month.	+	B	A	–f	–	Died of brain tumor
Craniopharyngioma	M; 7	2	4500/sellar	L hand mild numbness, clumsy for 8 months.	–	B	A	+	–	Dependent, stable
Medulloblastoma	F; 9	14	5000/p. fossa; 3600/CS	sudden L limb weakness	–	U	A	+	–	Died of brain tumor
Medulloblastoma	F; 9	20	5208/p. fossa; 3954/CS	Acute slurred speech	–	B	AP	–g	+	Dependent, stable
Craniopharyngioma	M; 10	3	5040/sellar	Noted in follow‐up MRI	–	B	AP	–f	–	Died of brain tumor
Craniopharyngioma	F; 2	2	5400/sellar	Sudden R hemiplegia, consciousness disturbance	–	U	A	–h	+	Dependent, deteriorated
Optic glioma	M; 7	3	5098/suprasellar	Sudden L hemiplegia, aphasia, R facial palsy	–	B	A	‐h	–	Dependent, stable

M, male; F, female; p, posterior; CS, craniospinal (axis); L, left; R, right; Sudden implies sudden onset; RT, radiotherapy; TIA, transient ischemic attack.

a + indicates that the patient was diagnosed with type‐1 neurofibromatosis.

b Unilateral/bilateral.

c Anterior/posterior circulation.

d + indicates that the patient received bypass surgery after moyamoya syndrome diagnosis.

e Independent: lives independently; stable: neurological function stable compared with the function prior to moyamoya syndrome; deteriorated: neurological function deteriorated compared with the function when first diagnosed with moyamoya syndrome. Patient is alive unless otherwise indicated.

f Bypass surgery not performed due to progressive brain tumor.

g Bypass surgery not performed due to TIA only.

h Bypass surgery not performed due to poor neurological condition.

The total follow‐up person‐years and number of patients with moyamoya vasculopathy by disease were, respectively, optic glioma (51, 2), craniopharyngioma (231, 3), astrocytoma (288, 1), and medulloblastoma (1023, 2). The incidence for each type of brain tumor after receiving RT, in person‐years, was thus 0.039 for optic glioma, 0.013 for craniopharyngioma, 0.003 for astrocytoma, and 0.002 for medulloblastoma.

**Figure 1:**
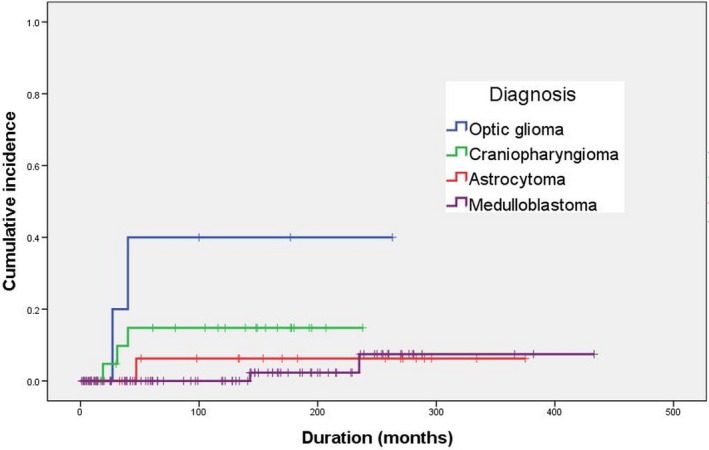
Cumulative incidence of postradiotheraphy moyamoya syndrome.

Based on the survival analysis, there was a significant difference (*P* = 0.002) in cumulative incidence of moyamoya vasculopathy in patients with different types of brain tumor after RT. The cumulative incidence of this condition by disease at 7 years was: optic glioma, 40% (standard error: 21.9%); craniopharyngioma, 14.8% (7.9%); astrocytoma, 6.2% (6.1%); and medulloblastoma, 0 (Fig. 1). The crude incidence of post‐RT moyamoya syndrome by diagnosis was: 2/6 (33.3%) for optic glioma, 3/24 (12.5%) for craniopharyngioma, 2/100 (2%) for medulloblastoma, and 1/19 (5.3%) for astrocytoma.

No patients died as a direct result of post‐RT moyamoya syndrome. There were three deaths: two patients died of recurrent tumor, and one died of adrenal crisis. In the three patients who underwent bypass surgery, the neurological function of the two remained stable compared with their function prior to developing moyamoya syndrome, and the other died of recurrent tumor. Two patients did not undergo bypass surgery because of poor disease prognosis. Bypass surgery was not performed in two patients because they only had TIAs. The reason for not performing bypass surgery for the remaining patient is unknown. Recurrent stroke occurred in two of the five patients not undergoing bypass surgery, causing deteriorated neurological function in one of them.

## Discussion

We estimated the incidence of post‐RT moyamoya syndrome in pediatric patients who had been treated for different types of primary brain tumors, finding that the incidence differed among diagnoses. Post‐RT moyamoya syndrome was not routinely fatal. However, repeated cerebral vascular events happened in those who did not undergo surgery.

Post‐RT moyamoya syndrome is a rare condition, and most publications on the subject are case reports or series that provide no estimate of incidence. This study is the first investigation on the incidence and long‐term outcome of this rare complication of RT. Given the ethnic homogeneity in Taiwan, the influence of ethnicity may make comparisons to other series challenging.

The etiology of moyamoya disease remains unknown. Known factors associated with moyamoya syndrome include sickle cell disease, NF1, therapeutic cranial irradiation, Down syndrome, congenital cardiac anomaly, renal‐artery stenosis, giant cervicofacial hemangiomas, and hyperthyroidism 3. A study based on 54 published cases of radiation‐induced moyamoya syndrome found that 29 (53.7%) arose following a diagnosis of optic glioma. This finding is consistent with our result that the highest incidence was found in those with optic glioma. The reason for the high incidence in this diagnosis may be from high‐dose radiation given to the suprasellar region, which contains circle of Willis. Moreover, optic glioma is associated with NF1. Patients with NF1 who received higher doses of radiation to the circle of Willis have increased risk of moyamoya syndrome development 11.

Craniopharyngioma arises inside the sella turcica. The circle of Willis would also be exposed to considerable scattered doses of radiation when this tumor is treated with RT, hence increasing the risk of complications. The only case of astrocytoma with post‐RT moyamoya syndrome was also the only one located in the suprasellar region, and that patient received a high dose of RT to the circle of Willis. Of the eight patients in the study, the high‐dose RT covered the suprasellar region in three and the sellar region in another three. However, in two cases of medulloblastoma, high‐dose RT was prescribed only to the posterior fossa surgical bed, and the radiation dose to the circle of Willis came only from low‐dose RT to the craniospinal axis. The latencies of moyamoya syndrome after RT in these two cases were longer than the other six cases. With the long‐term follow‐up in our study, we were able to identify a considerable incidence of moyamoya syndrome in those who only received low‐dose RT to the circle of Willis. Therefore, lower doses of radiotherapy might also induce moyamoya syndrome after a long latency.

In a study of pediatric patients with primary brain tumors treated at the Dana‐Farber Cancer Institute between 1990 and 2000, the crude incidence of post‐RT moyamoya syndrome was found to be 9/31 (29%) for optic glioma, 1/23 (4.3%) for craniopharyngioma, and 1/65 (1.5%) for medulloblastoma 11. Those data are not significantly different from those of this study. None of the patients in the Dana‐Faber study were of Asian descent, while all patients in this study were ethnic Taiwanese.

The crude incidence of post‐RT moyamoya syndrome in pediatric patients with craniopharyngioma was found to be 2/15 (13.3%) in a Korean series 8. That crude incidence is not different from the crude incidence found in this study.

Published data suggests that surgical revascularization is a safe intervention for pediatric moyamoya syndrome, and most treated patients derive some symptomatic benefit 20. In this study, a favorable outcome after bypass surgery was also found in patients with post‐RT moyamoya syndrome. Thus, surgical revascularization should be considered first for these patients. Based on this study, screening magnetic resonance angiography (MRA) on patients treated with RT, especially in cases of optic glioma and craniopharyngioma, may be worthwhile, for the high incidence and potential benefits of timely surgical intervention of early diagnosis.

### Limitation of the study

Only 69.3% of patients receiving radiation for brain tumors had complete notes and follow‐up imaging, potentially compromising the results. Only those patients with stroke or TIA were assessed for moyamoya syndrome, potentially omitting early cases, though one case was identified on routine imaging. Radiotherapy dose to the vasculature was not estimated, as we could not reliably estimate the scattered radiation dose from the two‐dimensional Cobalt‐60 plan.

## Conclusion

The incidence of post‐RT moyamoya syndrome in pediatric patients in Taiwan with each type of primary brain tumor was estimated. The incidence differed among diagnoses, with the highest incidence noted in optic glioma. The crude incidence was not different from those reported in two other series, from the United States and Korea. The clinical outcome is usually favorable. Stable long‐term neurological function may be achieved after timely surgical intervention.

## Conflict of Interest

None declared.
